# CD20-bearing extracellular vesicles are associated with prognostic biomarkers of patients with AIDS-NHL

**DOI:** 10.1038/s41598-025-11128-1

**Published:** 2025-07-12

**Authors:** Laura E. Martínez, Shelly Lensing, Di Chang, Larry I. Magpantay, Yu Guo, Ronald Mitsuyasu, Richard F. Ambinder, Joseph A. Sparano, Otoniel Martínez-Maza, Marta Epeldegui

**Affiliations:** 1 UCLA AIDS Institute and David Geffen School of Medicine, University of California, Los Angeles, CA USA; 2 Department of Obstetrics and Gynecology, David Geffen School of Medicine, University of California, Los Angeles, CA USA; 3 St. Jude Children’s Research Hospital, Memphis, TN USA; 4 University of Arkansas for Medical Sciences, Little Rock, AR USA; 5 Division of Hematologic Malignancies, Johns Hopkins Sidney Kimmel Comprehensive Cancer Center, John Hopkins University, Baltimore, MD USA; 6 Division of Hematology Oncology, Mount Sinai, NY USA; 7 Jonsson Comprehensive Cancer Center, University of California, Los Angeles, CA USA; 8 UCLA AIDS Institute, Biomedical Sciences Research Building, Rm. 173, Los Angeles, CA 90095 USA

**Keywords:** AIDS-associated non-Hodgkin lymphoma (AIDS-NHL), CD20, Anti-CD20, Rituximab, EPOCH, Extracellular vesicles, Tumour immunology, Prognostic markers

## Abstract

CD20 is a tetraspan membrane-bound protein vital for the development, activation, and differentiation of human B-cells. Non-Hodgkin lymphoma (NHL) is the most common hematological cancer in people living with (PLWH), and most B-cell NHLs express CD20. Extracellular vesicles (EVs) are nanosized vesicles released by cells into the extracellular environment. Here, we examined the association between plasma-derived CD20^+^ EVs and prognostic biomarkers in the AIDS Malignancy Consortium (AMC) 034 trial. We isolated EVs from plasma of patients with AIDS-NHL before and after initiation of treatment (rituximab plus concurrent infusional EPOCH), measuring CD20 on EVs by ELISA. Ramos cells were treated with EVs isolated from NHL and AIDS-NHL lymphoma cell lines in the presence of rituximab to measure if EVs protect cells from rituximab cytotoxicity. Baseline plasma levels of CD20^+^ EVs were significantly higher in non-responders and in patients with diffuse large B-cell lymphoma (DLBCL) with high International Prognostic Index (IPI) scores (2 to 3) compared to those with lower IPI scores (0 to 1). Moreover, baseline plasma levels of CD20^+^ EVs were significantly associated with baseline levels of prognostic biomarkers for AIDS-NHL. We further demonstrate that EVs from NHL and AIDS-NHL cell lines can sequester rituximab and inhibit apoptosis.

**Supplementary Information:**

The online version contains supplementary material available at 10.1038/s41598-025-11128-1.

## Background

Non-Hodgkin lymphoma (NHL) remains a significant cause of morbidity and mortality among people living with HIV (PLWH) in the post-antiretroviral therapy (ART) era^1,2^. Over 40% of PLWH are eventually diagnosed with AIDS-associated lymphomas^3^. Patients with AIDS-associated NHL (AIDS-NHL) may present with high grade, extranodal disease^3^. The most common subtypes of AIDS-NHL are diffuse large B-cell lymphoma (DLBCL) and Burkitt lymphoma (BL), accounting for 50% and 40% of cases, respectively^4,5^. Other subtypes, including primary central nervous system lymphoma (PCNSL), plasmablastic lymphoma (PBL), and primary effusion lymphoma (PEL), collectively make up the remaining 10%^4,5^.

Rituximab or other anti-CD20 biosimilar is part of standard first-line treatment for PLWH with CD20^+^ B-cell lymphomas. Typically, it is combined with etoposide, vincristine, doxorubicin, cyclophosphamide, and prednisone (EPOCH) chemotherapy or cyclophosphamide, doxorubicin, vincristine, and prednisone (CHOP)^6–10^. Studies show that rituximab containing regimens improve the overall survival of PLWH with CD20^+^ B-cell NHLs^6^. The clinical efficacy of rituximab has led to the development of other anti-CD20 monoclonal antibodies (i.e. obinutuzumab, ofatumumab, veltuzumab, and ocrelizumab)^11^.

Rituximab targets CD20 and can induce tumor killing via pro-apoptotic effects, complement-dependent cytolysis (CDC), and antibody-dependent cellular cytotoxicity (ADCC)^11^. Anti-CD20 therapy can also target the T-cell immune compartment^12–15^. CD20 expression is elevated in HIV-reactivated CD4^+^ T-cells, further rendering latent viral reservoir cells susceptible to the effects of rituximab^16^. HIV infected T-cells with high CD20 expression have also been identified in lymph nodes of PLWH^17–19^.

Extracellular vesicles (EVs) have the potential to serve as both diagnostic and predictive markers of cancer. EVs carry bioactive molecules such as lipids, metabolites, proteins, and nucleic acids, and can mediate intra- and intercellular communication at near and long-distance sites to promote cell growth and survival^20^. Emerging evidence shows that tumor-derived EVs can play a role in the development and progression of cancer^21^. Tumor-derived EVs participate in the formation and progression of different cancer processes, including invasion, angiogenesis, remodeling of the tumor microenvironment, metastasis, and drug-resistance, thus, creating an increasing challenge to cancer therapy^22^. Therefore, EVs are promising non-invasive correlates of tumor progression and are considered potential biomarkers for the diagnosis and prognosis of different types of cancer^22–28^.

We previously identified prognostic biomarkers with treatment response and survival in patients with AIDS-NHL enrolled in the AIDS Malignancy Consortium (AMC)−034 trial^29,30^. AMC-034 was a randomized phase 2 trial evaluating the safety and efficacy of infusional EPOCH chemotherapy administered either concurrently or sequentially with rituximab^9^. Eligible trial participants had previously untreated, histologically or cytologically confirmed aggressive CD20^+^ B-cell NHL, including DLBCL, BL, or other HIV-associated aggressive lymphomas^9^. Patients had stage II–IV disease (or stage I with elevated serum lactate dehydrogenase (LDH)), ECOG performance status of 0–2, and were 18 years of age and older^9^. Treatment led to reduced plasma levels of CXCL13, sCD27, and sCD30, lasting up to one year post-therapy^9^. In multivariate analyses, elevated pre-treatment IL-6 was linked to reduced overall survival (OS), and higher IL-10 to shorter progression-free survival (PFS). Kaplan-Meier analyses further showed that patients with CXCL13 or IL-6 levels above the median, or detectable IL-10, had poorer OS and PFS^9,29^. Recently, we quantified plasma levels of biomarkers related to B-cell activation and/or differerentiation (BAFF/BLyS IL-6, sCD23, sCD27, sIL-2Rα/sCD25, sCD30, CXCL13, CXCL10, sTNF-RII, sCD44, IL-10), macrophage activation (IL-18, CCL2/MCP-1, TNFα, CCL17/TARC, sCD163), and microbial translocation (sCD14, LBP)^29,30^. Post-treatment analysis revealed that TNFα, sIL-2Rα**/**sCD25, LBP, and TARC/CCL17 plasma levels significantly declined following rituximab plus infusional EPOCH chemotherapy^29,30^. Higher pre-treatment plasma levels of BAFF, sCD14, sTNF-RII, and CCL2/MCP-1 were univariately associated with worse OS, while elevated levels of BAFF, sTNF-RII, and CCL2/MCP-1 were also associated with shorter PFS^30^.

In the present study, we examined how baselinies plasma levels of CD20^+^ extracellular vesicles (EVs) relate to these previously characterized biomarkers of AIDS-NHL, such as IL-10, CXCL13, IL-10, BAFF, sCD14, sTNF-RII, sIL-2Rα**/**sCD25, IL-18, and CCL12-MCP-1^29–31^. Here, we found that CD20^+^ EVs were significantly associated with treatment response and were significantly elevated in plasma of trial participants with DLBCL tumor subtype and International Prognostic Index (IPI) scores of 2 to 3 compared to trial participants with scores of 0 to 1. Significant associations were observed between baseline plasma levels of CD20^+^ EVs and baseline plasma levels of prognostic biomarkers of AIDS-NHL: sCD27, CXCL13, sIL-2Rα**/**sCD25, sTNF-RII, sCD163, IL-18, LBP, and EndoCab IgM. Moreover, EVs isolated from cell culture supernatant of different NHL and AIDS-NHL cell lines carried CD20 and protected Ramos cells from rituximab-induced cytotoxicity. Thus, this study evaluates the prognostic and predictive value of pre-treatment CD20^+^ EVs in AIDS-NHL.

## **Methods**

### AIDS Malignancy Consortium (AMC) 034 study population

The AIDS Malignancy Consortium (AMC) trial, AMC protocol #034 (AMC-034) compared infusional combination chemotherapy (EPOCH: etoposide, vincristine, doxorubicin, cyclophosphamide, and prednisone) with concurrent or sequential rituximab^29^. Of the 106 patients with AIDS-NHL enrolled in the AMC-034 trial, plasma specimens were available from 57 patients with intermediate- or high-grade HIV-associated B-cell NHL (50 patients had DLBCL, 17 had Burkitt’s lymphoma, and 2 were classified only as lymphoma). Patient characteristics and clinical responses were defined in the report detailing AMC-034 trial results^29^. Briefly, the median age of lymphoma patients was 42.6 +/- 8.8 years. Lymphoma patients had a median HIV plasma level of 9,908 [inter-quartile range (IQR) between 492.5 and 45,660], and a median CD4^+^ T-cell count of 187 cells/mm^3^ (IQR between 82 and 333). Plasma was collected before the initiation of therapy, at the end of the first cycle (within a week or less), and at 6 months and one year after completion of treatment.

### Isolation of extracellular vesicles from plasma of AMC-034 trial participants

Plasma samples of 0.5 ml were differentially centrifuged at 2,000 x g for 20 min at room temperature to remove cell debris. The supernatant with clarified plasma was then transferred to a new tube and centrifuged at 10,000 x g for 20 min at room temperature to remove additional debris. The supernatant containing the clarified plasma was then transferred to a new tube with 0.5 ml volume of 1X PBS and mixed thoroughly, and 0.2 ml volume of the exosome precipitation reagent (from plasma) was added to the sample (Catalog No. 4484450; Invitrogen Total Exosome Isolation Kit (from plasma), Thermo Fisher Scientific, Waltham, MA, USA). The plasma/reagent solution was mixed by vortexing until the solution was homogenous and the samples were incubated at room temperature for 10 min. After the incubation period, samples were centrifuged at 10,000 x g for 5 min at room temperature to pellet exosomes. The supernatant was retrieved by pipet and discarded. The exosome pellet was centrifuged for an additional 30 s at 10,000 x g to collect any residual reagent. The residual supernatant was carefully discarded with a pipet. Lastly, 300 µl of 1X PBS was added to the pellet and resuspend with a pipet to disperse EVs for a homogeneous mixture. Isolated exosomes were stored at 4^o^C overnight or kept at −20^o^C until they were analyzed by Luminex multiplex immunometric assay or ELISA.

### CD20 ELISA

For detection of CD20 on EVs isolated from plasma of AMC-034 trial participants, we used 96-well ELISA plates coated with Human B-lymphocyte MS4A1 (CD20) ELISA Kit (Catalog No. MBS283766; MyBioSource, Inc., San Diego, CA, USA), according to the manufacturer’s instructions. Extracellular vesicle samples were retrieved from 4^o^C and left at room temperature for at least 20 min before loading on the ELISA plate. Each sample was diluted 1:2 in 1X PBS with 0.1% Tween-20 for a final concentration of 0.05% Tween-20 in PBS. Results were acquired from two independent reproducible readings at 450 nm from duplicate wells using a VersaMax Microplate Reader (Molecular Devices, LCC., San Jose, CA, USA) with SoftMax Pro Software (5.4) for Data Acquisition & Analysis (Molecular Devices, LCC., San Jose, CA, USA). The same ELISA platform and protocol was used for detection of CD20 on EVs isolated from supernatant of B-cell lymphoma cell lines.

### **Cell lines**

Human B-cell derived lymphoma cell lines of NHL (Raji, Ramos) and AIDS-NHL (2F7, OY6, R, and RRBL) were used in this study. Raji is a cell line from a Burkitt’s lymphoma (BL) (NHL) and was obtained from the American Type Culture Collection (ATCC) (Rockville, MD, USA). Ramos was derived from a patient with BL (NHL) and was obtained from the ATCC. The 2F7 cell line was derived from a patient with BL (AIDS-NHL) and was obtained from the ATCC^32^. The RRBL cell line was a gift of Dr. Steven Miles at UCLA and was derived from a tumor biopsy specimen of an AIDS-NHL patient with BL tumor subtype. The R cell line was isolated from a tumor biopsy specimen of an AIDS patient with aggressive lymphoma and with DLBCL tumor subtype and was provided by the laboratory of Dr. Zaki Salahuddin, as described^33^. OY6 is a lymphoblastoid cell line (LCL). An immortalized line of human T-lymphocyte cells (Jurkat) was used as a control non-B cell line when measuring CD20 expression on EVs by ELISA. Jurkat is a cell line established from the peripheral blood of a patient with acute T-cell leukemia and was obtained from the ATCC.

### Cell culture conditions and maintenance

All cell lines were maintained in RPMI-1640 medium (Gibco, Thermo Fisher Scientific, Waltham, MA, USA) supplemented with heat-inactivated 10% FBS and 1% penicillin-streptomycin (Gibco, Thermo Fisher Scientific, Waltham, MA, USA) at 37^o^C with 5% CO_2_. Cells were split every 2–3 days until ready to be used for extracellular vesicle isolation.

### Isolation of extracellular vesicles from cell lines

R, RRBL, Ramos, Raji, 2F7, OY6, and Jurkat cells (5 × 10^7^ to 1 × 10^8^) were cultured in serum-free media (AT-dBSA) for 24 h. After 24 h, cells and cell debris were removed by centrifugation at 2,000*g* for 30 min at room temperature. Total exosome isolation reagent was added to the cell-free and debris-free culture supernatant (Catalog No. 4478359; Invitrogen Total Exosome Isolation Kit (from cell culture media), Thermo Fisher Scientific, Waltham, MA, USA). For each 1 ml of cell culture supernatant, 0.5 ml of exosome isolation reagent was added, mixed, and incubated at 4 °C overnight. The next day, samples were centrifuged at 10,000 *g* for 1 h at 4^o^C. The exosome pellet was resuspended in 1X PBS (Gibco, Thermo Fisher Scientific, Waltham, MA, USA). Total protein level was measured using the Micro BCA protein assay (Thermo Scientific, Rockford, IL, USA) and absorbance was read at 562 nm using a Molecular Devices VersaMax microplate reader. Data was analyzed by SoftMax Pro software (5.4) (Molecular Devices LLC., San Jose, CA, USA). Total protein concentrations were adjusted for Western blot analysis.

### Western blot of extracellular vesicles isolated from cell lines

Extracellular vesicles were lysed by adding an equal volume of 1X RIPA lysis buffer (pH 7.4 +/- 0.1) with Protease Inhibitor Cocktail (in DMSO), including 200 mM PMSF solution (in DMSO) and 100 mM Sodium Orthovanadate solution (Catalog No. sc-24948; Santa Cruz Biotechnology Inc., Santa Cruz, CA, USA), and then incubated at 4^o^C for 15 min. Preparations were normalized for protein content and 20 µg of protein were prepared with 4X Protein Sample Loading Buffer (LI-COR Biosciences, Lincoln, NE, USA) for a final concentration of 1X, and then incubated at 70^o^C for 10 min. Samples were then loaded into each well of a pre-cast 4–12% Bis-Tris Mini Protein Gel (1.0 mm) (3.5 to 260 kDa) (Invitrogen, Carlsbad, CA, USA), run at 130 V, and then transferred to a Millipore Immobilin^®^-FL PVDF Membrane (0.45 μm) using the Mini-Protean Tetra System (Bio-Rad, Hercules, CA, USA) at 100 V for 1 h. Membranes were then immersed in 100% methanol for 30 s, rinsed once in double distilled water, and then rinsed in 1X Intercept^®^ TBS Blocking Buffer (1X TBS) (LI-COR Biosciences, Lincoln, NE, USA) for 5 min and blocked in 1X TBS for 1 h at room temperature with gentle shaking. Membranes were then incubated with the following primary antibodies: mouse monoclonal anti-human CD9 (Catalog No. 602.29 cl.11; Developmental Studies Hybridoma Bank, Iowa City, IA, USA) at 1:50 multiplexed with rabbit polyclonal anti-human TSG101 (Catalog No. 28283-1-AP; Proteintech, Rosemont, IL, USA) at 1:2,000; mouse monoclonal anti-human CD81 (clone 1D6) (Catalog No. NB10065805; Novus Biologicals, Centennial, CO, USA) at 1:100 multiplexed with rabbit polyclonal anti-human HSP70/HSPA1A (Catalog No. AF1663; R&D Systems, Minneapolis, MN, USA) at 1:1000; and anti-human CD63 (Catalog No. H5C6; Developmental Studies Hybridoma Bank, Iowa City, IA, USA). All primary antibodies were diluted in 1X TBS with 0.2% Tween-20 and incubated overnight at 4^o^C with gentle shaking. The next day, the blot was rinsed 3 times with 1X TBS-0.1% Tween-20 for 5 min each wash over a platform shaker, and then incubated with appropriate secondary antibodies: LI-COR IRDye 800CW goat anti-mouse IgG (H + L) secondary antibody at 1:10,000 (Catalog No. 926-32210) and/or LI-COR IRDye 680RD goat anti-rabbit IgG (H + L) secondary antibody at 1:10,000 (Catalog No. 926-68071; LI-COR Biosciences, Lincoln, NE, USA). Secondary antibodies were diluted in 1X TBS with 0.2% Tween-20 and 0.02% SDS for 1 h at room temperature with gentle shaking. The blot was then washed 3 times with 1X TBS-0.1% Tween-20 for 5 min each wash while shaking vigorously over a platform shaker at room temperature. Membranes were then rinsed with 1X TBS to remove residual Tween-20. Blots were then immediately scanned at 700–800 nm using a ChemiDoc™ Touch Imaging System (Bio-Rad, Hercules, CA, USA) at the UCLA AIDS Institute, and TIFF images were collected.

### Treatment of Ramos cells with extracellular vesicles isolated from NHL and AIDS-NHL cell lines and rituximab treatment

Ramos cells were plated at a density of 30,000 cells per well of a 96-well plate. Ramos cells were cultured in the presence of 30 µg of EVs (per cell line) and 3 µg/ml of anti-hCD20 (anti-hCD20-hIgG1) or rituximab monoclonal antibody (Catalog No. hcd20-mab1; InvivoGen, San Diego, CA, USA) in complete RPMI medium for 48 h at 37^o^C with 5% CO_2_. Cells were stained with Annexin V and Propidium Iodide using the Annexin V Ready Flow Conjugates for Apoptosis Detection (Alexa Fluor 488) according to manufacturer’s instructions (Catalog No. R37174; Invitrogen, Thermo Fisher Scientific, Waltham, MA, USA) and analyzed for flow cytometry to evaluate subpopulations of cells undergoing apoptosis using a BD LSR Fortessa™ cell analyzer. Data was then analyzed with the FCS Express software program (v7.04.0004, De Novo Software, Pasadena, CA, USA). The fold increase of apoptosis was determined by calculating the ratio of percent double positive cells (PI + and Annexin V+) after treatment with rituximab over the untreated Ramos cell condition.

### Detection of plasma biomarker levels in the AMC-034 trial

We analyzed previously acquired data to assess the relationship between CD20^+^ EVs in plasma and biomarkers of AIDS-NHL; these plasma biomarker results were reported in a prior publication^30^. Briefly, plasma levels of EndoCab IgM were determined by ELISA (Hycult Biotech, Uden, The Netherlands), according to the manufacturers’ instructions. Plasma levels of sCD14, sCD27, LBP, FABP2, IL-18, CCL2/MCP-1, sCD163, CXCL10/IP-10, CCL17/TARC, CXCL13, TNF-α, BAFF/BLyS, sTNF-RII, sCD44, and sIL-2Rα/sCD25 were determined using the Luminex multiplex assay platform with custom-made panels as previously described^30^.

#### Statistical analysis

The pre-treatment versus post-treatment comparison of plasma-derived CD20^+^ EVs were made using the paired, nonparametric Wilcoxon signed-rank test. The relationships between CD20^+^ EVs and plasma levels of biomarkers associated with AIDS-NHL were assessed by determining Spearman’s rank correlation coefficients. Relationships with participant characteristics (CD4^+^ T-cell number, HIV viral load, IPI score) were evaluated with correlations or non-parametric Wilcoxon rank-sum tests. The International Prognostic Index (IPI) is a clinical tool used to predict prognosis in patients with aggressive non-Hodgkin lymphoma, including DLBCL, the most common subtype of AIDS-associated NHL^9^. In AIDS-NHL, IPI is used for stratifying patients by risk to predict overall survival (OS), progression-free survival (PFS), and response to therapy. Its predictive power can be affected by CD4 count, HIV viral load, adherence to ART, and any potential co-infections in people living with HIV. For survival endpoints (OS, PFS), Kaplan-Meier estimates were calculated according to CD20 group (defined by the median split for CD20-expressing EVs) and comparisons were made using log-rank tests: for Complete Response (CR) according to CD20 group, comparisons were made using Fisher’s exact test. As there are no normal ranges for these biomarkers, median cut points were used for ease of interpretation. The association between CD20^+^ EVs with response status (Non-Response (NR), Partial Response (PR), and Complete Response (CR)) was assessed using the Kruskal-Wallis test. Relationships between CD20^+^ EVs and plasma levels of prognostic biomarkers of AIDS-NHL were assessed by determining Spearman’s rank correlation coefficients. *P*-values were not adjusted for multiple comparisons for this exploratory study. In all cases, a two-tailed value of *p* < 0.05 was considered statistically significant.

## Results

### **Baseline levels of CD20**^**+**^**EVs significantly correlate with treatment response**

In this study, we evaluated whether CD20^+^ EVs are present in the peripheral blood of AIDS-NHL trial participants enrolled in the AMC-034 trial. Extracellular vesicles were isolated from plasma of AIDS-NHL trial participants at pre-treatment or baseline (*N* = 53) and post-treatment (*N* = 37) with rituximab and concurrent infusional combination chemotherapy (EPOCH) and CD20 expression on EVs was measured by ELISA. Although CD20^+^ EV levels trended to be lower at post-treatment, we did not find significant differences between matched pre- (*N* = 37) and post-treatment (*N* = 37) levels (median CD20 value at pre- (2.22 ng/ml) and post-treatment (2.08 ng/ml); *p* = 0.063, Wilcoxon signed-rank test).

**Fig. 1:**
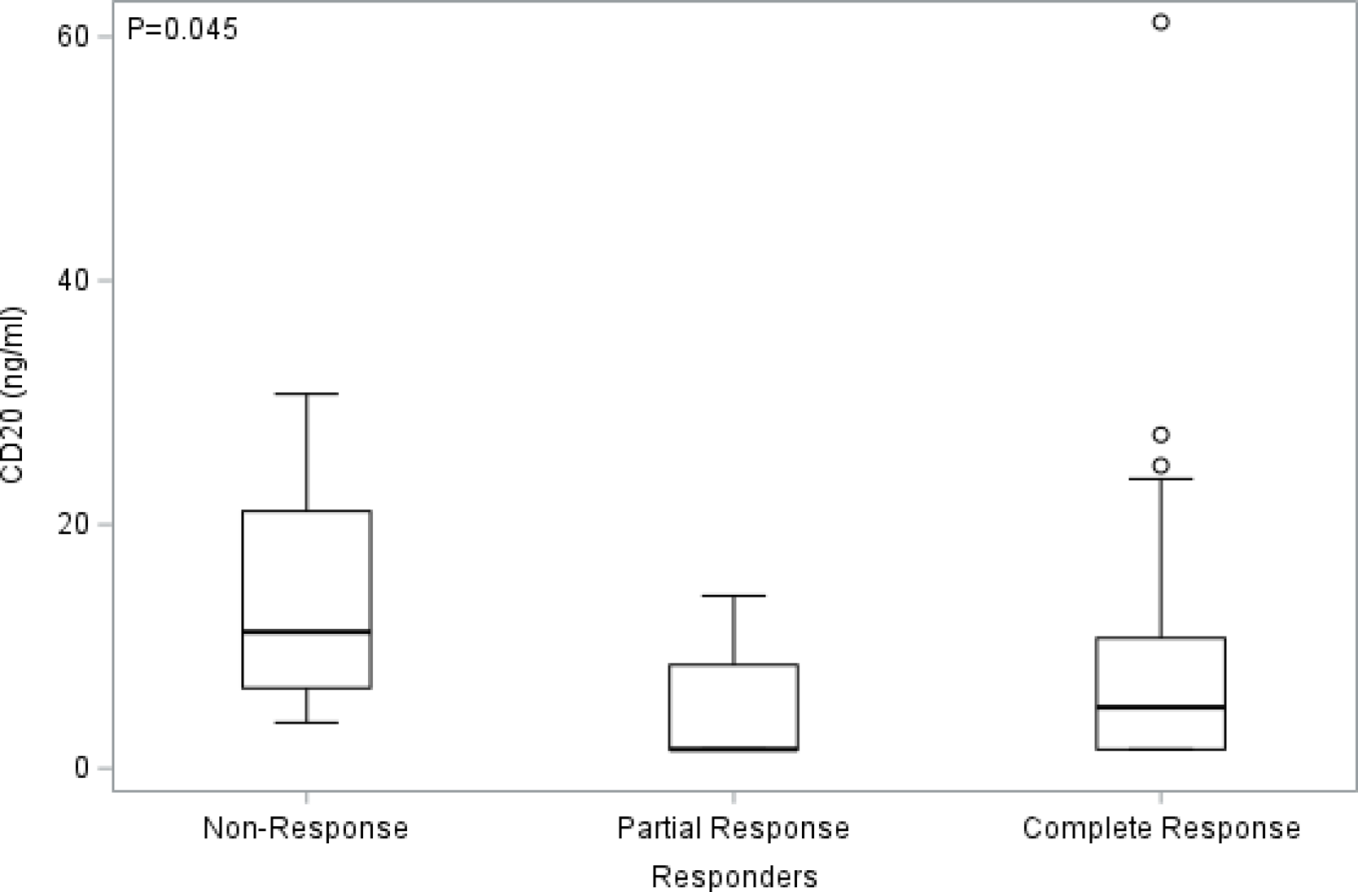
**Partial and complete responders show significantly reduced levels of CD20**^**+**^ **EVs in circulation at baseline.** Comparison of baseline (pre-treatment) biomarker response changes in the levels of CD20^+^ EVs in plasma of AMC-034 trial participants with Non-Response (NR) (*N* = 7), Partial Response (PR) (*N* = 12), or Complete Response (CR) (*N* = 34) status to therapy. Shown are the distributions of CD20 concentrations in EVs by response and median values are indicated by a line. Statistical comparisons were made between CD20-expressing EVs in NR vs. PR vs. CR using a Kruskal-Wallis test, where *p* = 0.045.

We then determined associations between baseline plasma levels of CD20^+^ EVs and treatment response to rituximab and EPOCH chemotherapy. Fifty-three participants with pre-treatment biomarker data and CD20^+^ EV data was evaluated, of whom 7 had non-response status (NR), 12 partial response (PR), and 34 complete response (CR). We found that baseline plasma levels of CD20^+^ EVs were significantly different with non-responders having higher plasma levels of CD20^+^ EVs and trial participants with partial response and complete response having lower levels (median, 1.6 ng/ml (PR) vs. 5.0 ng/ml (CR) vs. 11.2 ng/ml (NR), *p* = 0.045, Kruskal-Wallis test) (Fig. 1). However, in a related analysis when baseline plasma levels of CD20^+^ EVs were dichotomized based on their median value, CD20 concentrations (ng/ml) did not significantly correlate with CR rates (*p* > 0.999) (Table 1). Both CD20 groups (< median and *≥* median) had 64% complete response rates. In addition, no significant difference was observed between CD20 groups for overall survival (OS) (*p* = 0.710) or progression-free survival (PFS) (*p* = 0.675, two-sample log-rank test) (Table 1).

**Table 1:** Relationship between baseline plasma levels of CD20^+^ EVs and outcome measures of AMC-034 trial participants.

Factor	*N*	Complete response rate (%)	*N*	1-Year OS (%) (95% CI)	1-Year PFS (%) (95% CI)
CD20^+^ EVs					
< Median	25	64	29	78.2 (57.7–89.6)	68.0 (47.5–81.9)
*≥*Median	28	64	29	75.7 (55.7–87.6)	75.0 (50.0–88.7)
OR/HR (95%CI)^c^		1.01 (0.33–3.12)		1.18 (0.49–2.86)	1.19 (0.53–2.67)
*p*		> 0.999^a^		0.710^b^	0.675^b^

Note: Median (IQR) plasma levels of CD20^+^ EVs were 4.9 (1.6–10.7), *N* = 58.

^a^Fisher’s exact test.

^b^Log-rank test.

^c^Odds Ratio (OR) and 95% confidence interval for complete responses: Hazard Ratio (HR) and 95% confidence interval for overall survival (OS) and progression-free survival (PFS) (unadjusted).

**Fig. 2:**
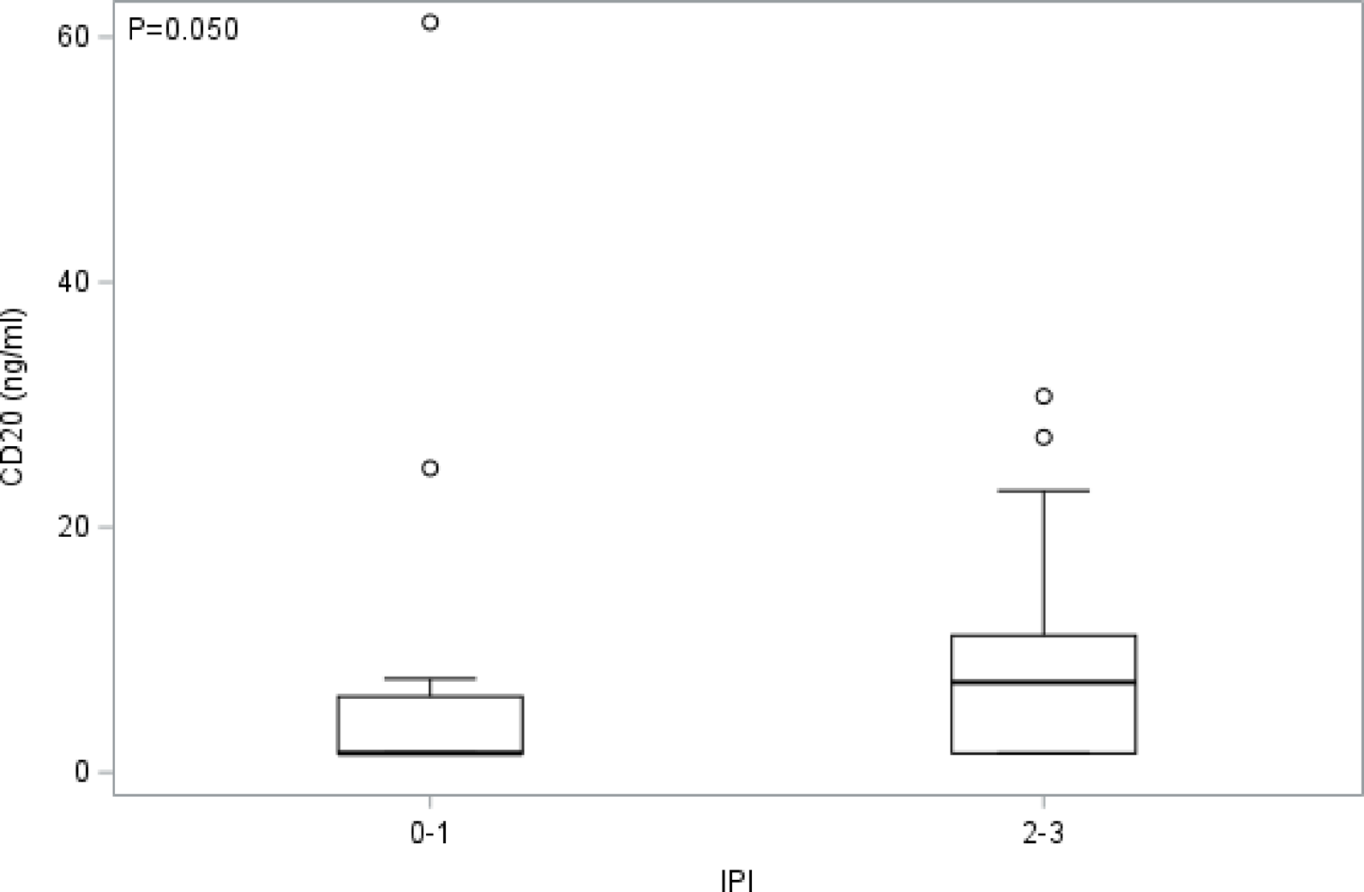
** Baseline plasma levels of CD20**^**+**^ **EVs are significantly elevated in AMC-034 trial participants with DLBCL tumor subtype and IPI scores of 2 to 3.** The box plot shows the distribution of CD20 concentration (ng/ml) on EVs according to IPI scores 0 to 1 (*N* = 18) and 2 to 3 (*N* = 29) for DLBCL tumor subtype. A two-sided Wilcoxon two-sample test was conducted, where *p* = 0.050.

The most common subtypes of AIDS-NHLs are diffuse large B-cell lymphoma (DLBCL) and Burkitt’s lymphoma (BL)^4,5^. For this study, we had CD20 EV data for 47 patients with DLBCL, 10 with BL, and 1 patient with follicular B-cell lymphoma. Because most patients had DLBCL tumor subtype, and given the small sample size for BL, we only evaluated relationships between CD20^+^ EVs and outcome measures for DLBCL. We found that dichotomized baseline plasma levels of CD20^+^ EVs did not significantly correlate with CR rates (*p* > 0.999), OS (*p* = 0.881), or PFS (*p* = 0.814) (**Supplemental Table **1). However, we did observe elevated plasma levels of CD20^+^ EVs at baseline and in circulation of DLBCL patients with IPI scores of 2 to 3 as compared to patients with IPI scores of 0 to 1 (**Fig. **2).

### **CD20**^**+**^**EVs correlate with baseline HIV viral load and prognostic biomarkers of AIDS-NHL**

We found that baseline plasma levels of CD20^+^ EVs significantly correlate with HIV viral load (*N* = 34; Spearman correlation coefficient = 0.42; *p* = 0.015), but not with CD4^+^ T-cell count (*N* = 36; Spearman correlation coefficient = −0.29; *p* = 0.097) (Table 2). We also determined significant positive associations between plasma levels of CD20^+^ EVs and plasma biomarker levels of sCD27, CXCL13, sIL-2Rα**/**sCD25, sTNF-RII, sCD163, IL-18, and LBP (Table 2). CD20^+^ EVs negatively correlated with plasma levels of EndoCab IgM (Spearman correlation coefficient = −0.44, *p* < 0.001) (Table 2). When we evaluated associations between post-treatment plasma levels of CD20^+^ EVs with post-treatment plasma levels of biomarkers, we found that BAFF was the only biomarker that significantly correlated with plasma levels of CD20^+^ EVs in specimens collected post-treatment (*N* = 29; Spearman correlation coefficient = 0.45, *p* = 0.014).

**Table 2:** Correlation measures between baseline plasma levels of CD20^+^ EVs and prognostic biomarkers of AIDS-NHL.

CD20^+^EVs	
**CD4**^**+**^ **T-cell count**	
Spearman ρ	0.28
*p* ^b^	0.097
N^c^	36
**HIV viral load**	
Spearman ρ	0.42
*p* ^b^	0.015
N^c^	34
**B-cell stimulatory molecules**	
**BAFF/BLyS**	
Spearman ρ	0.15
*p*	0.276
N	58
**sCD27**	
Spearman ρ	0.46
*p*	0.004
N	36
**CXCL13**	
Spearman ρ	0.36
*p*	0.026
N	37
**sIL-2Rα/sCD25**	
Spearman ρ	0.38
*p*	0.003
N	58
**sTNF-RII**	
Spearman ρ	0.41
*p*	0.001
N	58
**FABP2**	
Spearman ρ	−0.13
*p*	0.334
N	58
**CCL2/MCP-1**	
Spearman ρ	0.04
*p*	0.793
N	58
**sCD44**	
Spearman ρ	0.07
*p*	0.609
N	58
**CCL17/TARC**	
Spearman ρ	0.12
*p*	0.355
N	58
**CXCL10/IP-10**	
Spearman ρ	0.20
*P*	0.139
N	58
**TNF-α**	
Spearman ρ	0.01
*p*	0.915
N	58
**Biomarkers of macrophage activation**	
**sCD14**	
Spearman ρ	0.16
*p*	0.230
N	58
**sCD163**	
Spearman ρ	0.43
*p*	0.001
N	58
**IL-18**	
Spearman ρ	0.42
*p*	< 0.001
N	58
**Biomarkers of microbial translocation**	
**LBP**	
Spearman ρ	0.33
*p*	0.011
N	58
**EndoCab IgM**	
Spearman ρ	−0.44
*p*	< 0.001
N	58

### **Extracellular vesicles from NHL and AIDS-NHL cell lines carry CD20 and sequester rituximab**,** inhibiting apoptosis**

**Fig. 3:**
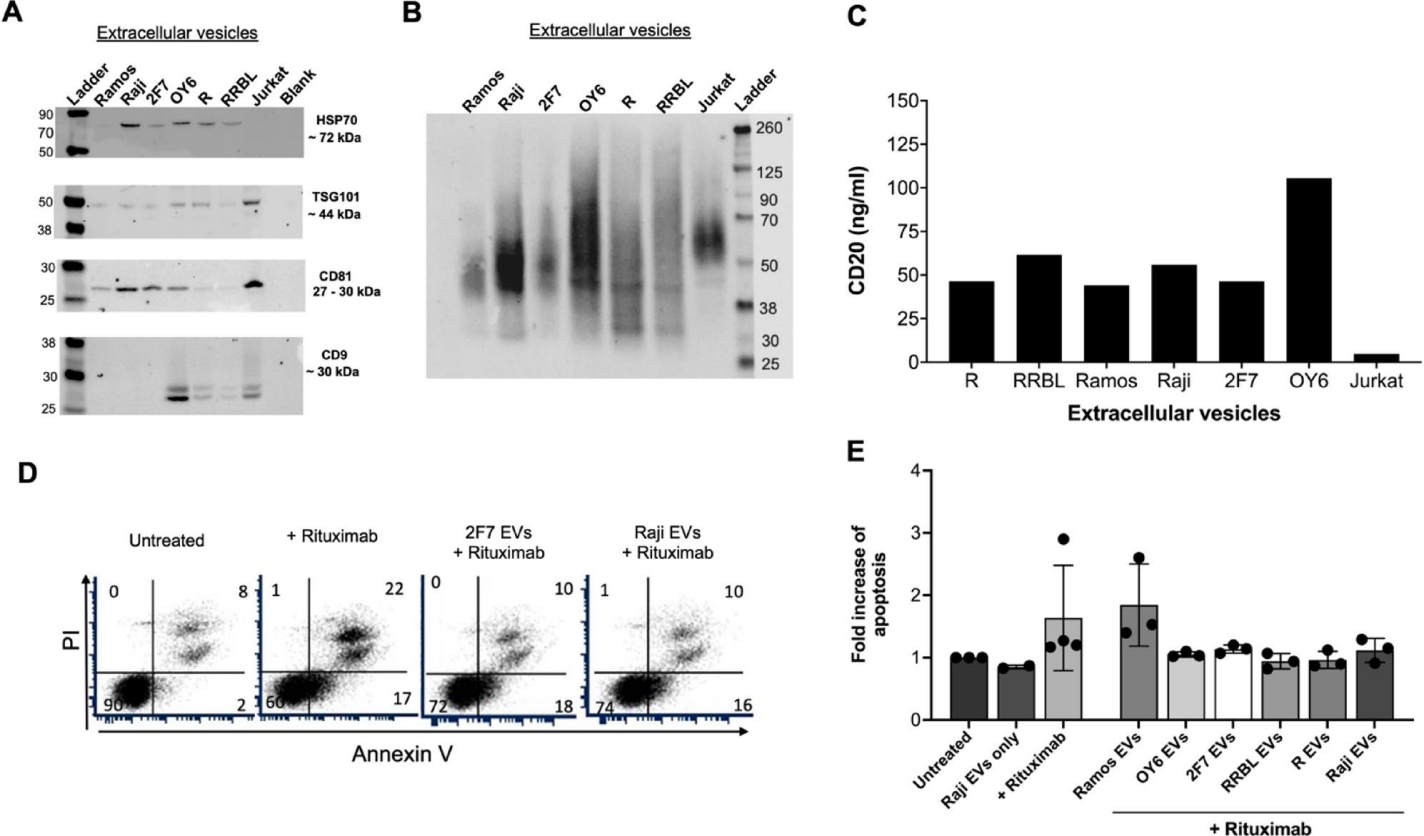
**EVs isolated from NHL and AIDS-NHL cell lines sequester rituximab and inhibit apoptosis in Ramos cells. (A)** Characterization of extracellular vesicles isolated from NHL and AIDS-NHL cell lines. Western blots of EVs demonstrate the presence of classic exosome markers: HSP70, TSG101, CD81, CD9, and (**B**) CD63. 20 µg of EV protein lysate was loaded into each well. Imaging/exposure times of each blot: CD81, 60 s; HSP70, 75 s; CD9, 60 s; TSG101, 40 s; and CD63, 60 s. **(C)** CD20 concentrations measured in EVs by ELISA. Data is for one Luminex assay run. **(D)** Ramos cells were treated with rituximab (3 µg/ml) in the presence or absence of EVs isolated from NHL (Raji, Ramos) or AIDS-NHL cell lines (2F7, RRBL, R) cell lines, including OY6, a lymphoblastoid cell line of AIDS-NHL. 30 µg of total EV protein was used for each treatment. Shown are representative flow cytometry plots of Ramos cells double stained with Propidium iodide (PI) and Annexin V-FITC after treatment with rituximab in the presence of 2F7 EVs or Raji EVs to evaluate subpopulations of cells undergoing apoptosis. Percentage values are shown in each quadrant of each plot. **(E)** Quantitative data presented as bar graphs showing the fold increase of apoptosis: Ratio of % double positive cells (PI + and Annexin V+) after treatment with rituximab/untreated Ramos cells. Results are from two to four independent experiments.

EVs may present CD20 on their surface and act as decoys to trap anti-CD20 rituximab^34–37^. Here, we isolated EVs released into the supernatant of B-cell lymphoma cells derived from NHL (Ramos and Raji) or AIDS-NHL (2F7, R, and RRBL) cases, and a lymphoblastoid cell line, OY6. EV lysates were analyzed for the expression of classical exosome markers by Western blot. HSP70, TSG101, CD81, and CD63 were confirmed in EV lysates (Fig. 3A-B). However, EVs isolated from supernatant of Ramos, Raji, and 2F7 cell lines did not express CD9 (Fig. 3A). Original blots are presented in **Supplementary Fig. 1**. We then measured CD20 concentration on the EVs by ELISA. EVs isolated from each cell line expressed CD20 but EVs isolated from OY6 showed the highest CD20 concentration (105 ng/ml) (Fig. 3C). EVs isolated from Jurkat cells, a T-cell lymphoma cell line included as a negative control, did not express CD20 (Fig. 3C).

Ramos cells were then treated with rituximab (3 µg/ml) in the presence or absence of EVs isolated from Ramos, OY6, 2F7, RRBL, R, or Raji cells (30 µg of total EV protein from each) for 48 h. Ramos cells were then double stained with propidium iodide and Annexin-V to determine subpopulations of cells undergoing apoptosis (Fig. 3D). We found that EVs isolated from OY6, 2F7, RRBL, R, or Raji were able to inhibit rituximab induced apoptosis in Ramos cells (Fig. 3E).

## Discussion

In this study, we measured plasma levels of CD20^+^ EVs and compared those concentrations with clinical and pathologic features of AIDS-NHL trial participants part of the AMC-034 trial, such as HIV viral load, CD4^+^ T-cell count, response to treatment, and patient outcome measures, including overall survival and progression-free survival. Both partial and complete responders had notably lower levels of CD20^+^ EVs compared to non-responders. This analysis examined baseline CD20^+^ EV concentrations as a continuous variable across the three response categories. When we examined pre-treatment levels of CD20^+^ EVs as a grouped variable to two response groups CR vs. PR/NR, we found that dichotomized CD20^+^ EV levels did not reveal correlations with overall survival or progression-free survival. While CD20^+^ EVs might not predict long-term outcomes, further research is needed to understand their biological complexity and role in lymphoma, including their interactions with AIDS-NHL biomarkers.

Evidence suggests that EVs play an important role in hematological malignancies and diffuse large B-cell lymphoma (DLBCL) development and progression^34,38,39^. We sought to compare plasma levels of CD20^+^ EVs between patients with DLBCL (*N* = 47) and Burkitt lymphoma (BL) (*N* = 10). Due to insufficient data from BL patients for meaningful analysis, we focused these studies on DLBCL patients. We observed that those with DLBCL and IPI scores of 2 (low intermediate) to 3 (high intermediate) had elevated baseline levels CD20^+^ EVs further suggesting increased risk of disease progression.

In this study, we found a significant correlation between baseline plasma levels of CD20^+^ EVs and baseline HIV viral load of patients with AIDS-NHL enrolled in the AMC-034 trial. Furthermore, CD20^+^ EVs showed significant positive correlations with potential prognostic biomarkers of AIDS-NHL^29,30,40,41^ at baseline: sCD27, CXCL13, sIL-2Rα/sCD25, sTNF-RII, sCD163, IL-18, LBP, and EndoCab IgM.

CXCL13 signals through the CXCR5 receptor and promotes the formation of secondary lymphoid tissues and directs B-cell and T follicular helper cell (Tfh) migration to germinal centers, leading to B-cell differentiation into plasma and memory B-cells^42^. Thus, CXCL13 is considered a general biomarker for germinal center activity. In PLWH, elevated plasma levels of CXCL13 correlate with advanced progression to AIDS^43–45^. In addition, increased CXCL13 production in HIV-infected lymph nodes may contribute to the downregulation of CXCR5^46,47^.

IL-18 is a pro-inflammatory cytokine produced by various cells, including B-cell, T-cells, monocytes, macrophages, dendritic cells, and non-hematopoietic cells like keratinocytes, mesenchymal cells, and astrocytes^48^. Our previous studies showed that EVs bearing CD40, CD40L, TNF-RII, or IL-6Rα significantly correlated with plasma levels of IL-18 in patients with AIDS-NHL from the AMC-034 trial^31^. Elevated levels of IL-18 may indicate a more aggressive disease or worse prognosis in NHL by affecting tumor growth and survival of tumor cells. Moreover, the significant correlation between baseline CD20^+^ EVs and soluble CD163 or sCD163, the extracellular portion of the CD163 macrophage marker, indicates increased macrophage activation and inflammation. During HIV infection, inflammatory stimuli can elevate sCD163 levels by inhibiting CD163 production and promoting its release from activated macrophages^49–51^. B-cell turnover and CD20^+^ EV secretion may be related to inflammatory responses against HIV involving macrophages.

TNF-RII is a receptor for tumor necrosis factor-alpha (TNF-α), a pro-inflammatory cytokine. Chronic HIV infection and AIDS are associated with elevated levels of soluble forms of TNF receptor superfamily (TNFRS) proteins, including sTNF-RII^52,53^. Our previous research linked baseline sTNF-RII levels with overall survival and progression-free survival in patients with AIDS-NHL from the AMC-034 trial^30^. In this study, CD20^+^ EVs did not significantly associate with overall survival or progression-free survival. However, the significant correlation between baseline CD20^+^ EVs and sTNF-RII suggests a relationship that may influence treatment response. Further investigation of CD20^+^ EVs and TNF-RII^+^ EVs could provide valuable insight into disease processes and treatment response in patients with AIDS-NHL. Additionally, we have shown that TNF-RII^+^ EVs significantly correlate with HIV viral load and baseline plasma levels of soluble TNF-RII, including biomarker levels of IL-6, IL-10, CXCL13, sCD25, IL-18, BAFF, sCD14, sCD44, and CXCL10^31^.

The lipopolyssacharide-binding protein (LBP) recognizes bacterial endotoxins like lipopolysaccharide (LPS), while EndoCab IgM detects endotoxin core antibodies in human plasma or serum, making them important biomarkers of microbial translocation. Microbial products such as LPS can trigger inflammation and immune activation by stimulating toll-like receptor (TLR-4) on monocytes^54^. The CD14 receptor on monocytes and macrophages binds LPS, triggering immune activation and inflammation^55^. When these cells are activated, CD14 is cleaved and released into the bloodstream as soluble CD14 (sCD14). Higher plasma levels of sCD14 and LPS have been observed in PLWH compared to HIV-negative individuals^54,56,57^. Our previous work showed that plasma levels of LBP and sCD14 were associated with AIDS-NHL risk in PLWH from the Multicenter AIDS Cohort Study (MACS) (now the MACS/WIHS Combined Cohort Study or MWCCS) before their AIDS-NHL diagnosis^40^. The correlation between plasma CD20^+^ EVs and LBP or EndoCab IgM suggests that these EVs are related to broader inflammatory or acute-phase responses associated with microbial translocation in patients with AIDS-NHL. Studies show that LPS-positive EVs are present in peripheral blood circulation of treatment naïve PLWH^58^. Future studies can explore whether CD20^+^ EVs carry LBP and other markers of microbial translocation, including bacterial LPS.

Aung et al.. found that exosomes isolated from aggressive B-cell lymphoma cell lines and primary lymphoma cells carried CD20, bound the anti-CD20 monoclonal antibody rituximab, consumed complement, and depleted soluble antibody in vitro, thereby protecting target cells from antibody-mediated attack^34^. They suggest that exosomes bearing CD20 act as a decoy, thus, reducing the number of rituximab molecules reaching tumor cells^34^. In this study, we examined CD20 expression on EVs released by NHL and AIDS-NHL cell lines, as well as a B-cell lymphoblastoid cell line. We found that EVs from both NHL and AIDS-NHL cells potentially bind rituximab in vitro and inhibit rituximab-induced apoptosis in Ramos cells. Therefore, it is plausible that CD20^+^ EVs may interact with rituximab in vivo, contributing to immune escape, tumor progression, and potentially driving therapeutic resistance in the tumor microenvironment.

Further research will explore how CD20^+^ EVs interact or influence biomarkers of AIDS-NHL risk and determine if CD20^+^ EVs display a functional role in modulating the levels of these biomarkers. Understanding these relationships could provide deeper insights into lymphoma pathology and treatment mechanisms. Although further validation is needed to establish the clinical utility of CD20^+^ EVs, they could potentially be used to refine diagnostic or prognostic assessments in AIDS-NHL. Moreover, future studies could investigate the immunomodulatory effects of EVs carrying HIV cargo. HIV-infected cells release exosomes containing viral RNA and proteins^59–64^, which can be transported to other cells in the microenvironment and influence pathogenesis and disease progression in AIDS-NHL. Thus, it will be crucial to assess the predictive value of CD20^+^ EVs through gene-expression profiling and proteomics. For example, research shows that exosomes isolated from plasma of B-cell lymphoma patients carry mRNA of oncogenes (cMYC, BCL-6, BCL-XL), the tumor suppressors PTEN, and important signaling pathway molecules (NF-kβ, AKT) dysregulated in NHL, which may serve as prognostic markers of lymphomagenesis^65^.

Moreover, future studies should focus on the proteomic profiling of plasma-derived EVs from patients with AIDS-NHL to identify additional immunoregulatory molecules linked to B-cell activation and/or dysfunction. Research should also characterize other features of CD20^+^ EVs by examining their cargo (i.e. RNA, mRNAs, microRNAs, DNA), proteomes, antigen display, and immunoglobulin expression. Additionally, evaluating the prognostic value of CD20^+^ EVs in patients with refractory disease or poor treatment response is essential.

Extracellular vesicles reflect the landscape of the tumor microenvironment (TME) and carry molecular signals from various cell types and not just malignant B-cells, including plasma cells, malignant T-cells, tumor-associated macrophages (TAMs), dendritic cells, natural killer cells, myeloid-derived suppressor cells (MDSCs), fibroblasts, endothelial cells, and mesenchymal stem cells (MSCs)^66,67^. The interactions between these cells and the EVs they secrete can significantly impact NHL progression and treatment response. The TME plays a crucial role in supporting tumor growth, immune evasion, and therapy resistance^66^. Therefore, profiling exosomal proteomes from different bodily fluids and tumor tissue is essential. In addition, tissue biopsies from patients with AIDS-NHL can be analyzed using imaging mass cytometry (IMC) to visualize EV localization within the tumor microenvironment.

## Limitations of the study

One of the main limitations of this study is the limited size sample of patients with BL compared to DLBCL tumor subtype. A larger cohort of patients with AIDS-NHL with BL would add power to the analysis. Our findings suggest limited or stong associations between basline dichotomized CD20^+^ EV levels and long-term clinical outcomes. However, continuous variable analyses support a potential inverse relationship between CD20^+^ EV levels and initial treatement response. Moreover, in this study we examine correlations with biomarkers previously identified in patients with AIDS-NHL enrolled in the AMC-034 trial. It would be important to expand these studies to other patient cohorts and to examine other biomarkers associated with response or survival in lymphoma.

## Conclusions

Baseline plasma levels of extracellular vesicles bearing CD20 are associated with plasma levels of biomarkers of B-cell activation and differentiation (sCD27, CXCL13, sIL-2Rα/sCD25, sTNF-RII), macrophage activation (sCD163, IL-18), and microbial translocation (LBP, EndoCab IgM) in patients with AIDS-NHL. The significant correlations between CD20^+^ EVs and these biomarkers of AIDS-NHL could be leveraged to develop comprehensive prognostic assessments. Because these correlations are at baseline, they could provide insight into the immune or inflammatory state of the patient before treatment and could be useful for tailoring treatment strategies. These findings have relevance in PLWH and patients with AIDS-NHL with high levels of tumor cells secreting extracellular vesicles while undergoing treatment with anti-B-cell therapy drugs, such as rituximab, and/or chemotherapy agents. Future studies should explore how CD20^+^ EVs might interfere with CD20-targeted therapies and assess the effectiveness of new bispecific antibodies, such as mosunatuzumab, glofitimab, and epcoritimab. These antibodies can bind CD3 on T-cells and CD20 on B-cells, potentially improving the targeting and killing of CD20^+^ B-cells.

## Electronic supplementary material

Below is the link to the electronic supplementary material.

Supplementary Material 1

## Data Availability

The datasets used and/or analyzed in the current study are available from the corresponding author on reasonable request and AIDS Malignancy Consortium (AMC) approval.
