# Supplementary material for: CD20-bearing extracellular vesicles are associated with prognostic biomarkers of patients with AIDS-NHL
Source: Sci Rep. 2025 Jul 12;15:25181. doi: 10.1038/s41598-025-11128-1 (PMC12254413; doi:10.1038/s41598-025-11128-1)
Supplement: Supplementary file 1 — Supplementary Material 1 [file 41598_2025_11128_MOESM1_ESM.docx]

**SUPPLEMENTAL MATERIAL**

**Supplemental Table 1**. **Relationship between baseline (pre-treatment) plasma levels of CD20^+^ EVs and outcome measures of AMC-034 trial participants with DCBCL.**

| **Factor** | **N** | **Complete response**  **rate (%)** | **N** | **1-Year OS (%) (95% CI)** | **1-Year PFS (%) (95% CI)** |
| --- | --- | --- | --- | --- | --- |
| **CD20^+^ EVs** |  |  |  |  |  |
| <Median* | 21 | 57 | 24 | 77.7 (54.3 – 90.1) | 65.5 (42.6 – 81.0) |
| >Median* | 23 | 59 | 23 | 78.0 (55.0 – 90.2) | 69.6 (46.6 – 84.2) |
| OR/HR (95%CI)^c^ |  | 1.08 (0.32 – 3.64) |  | 1.08 (0.42 – 2.80) | 1.12 (0.48 – 2.64) |
| *p* |  | > 0.999^a^ |  | 0.881^b^ | 0.814^b^ |

^a^Fisher’s exact test.

^b^Log-rank test.

^c^OR and 95% confidence interval for complete response; Hazard Ratio (HR) and 95% confidence interval for overall survival (OS) and progression-free survival (PFS) (unadjusted).

* The median values are based on the full dataset (N = 58).

**
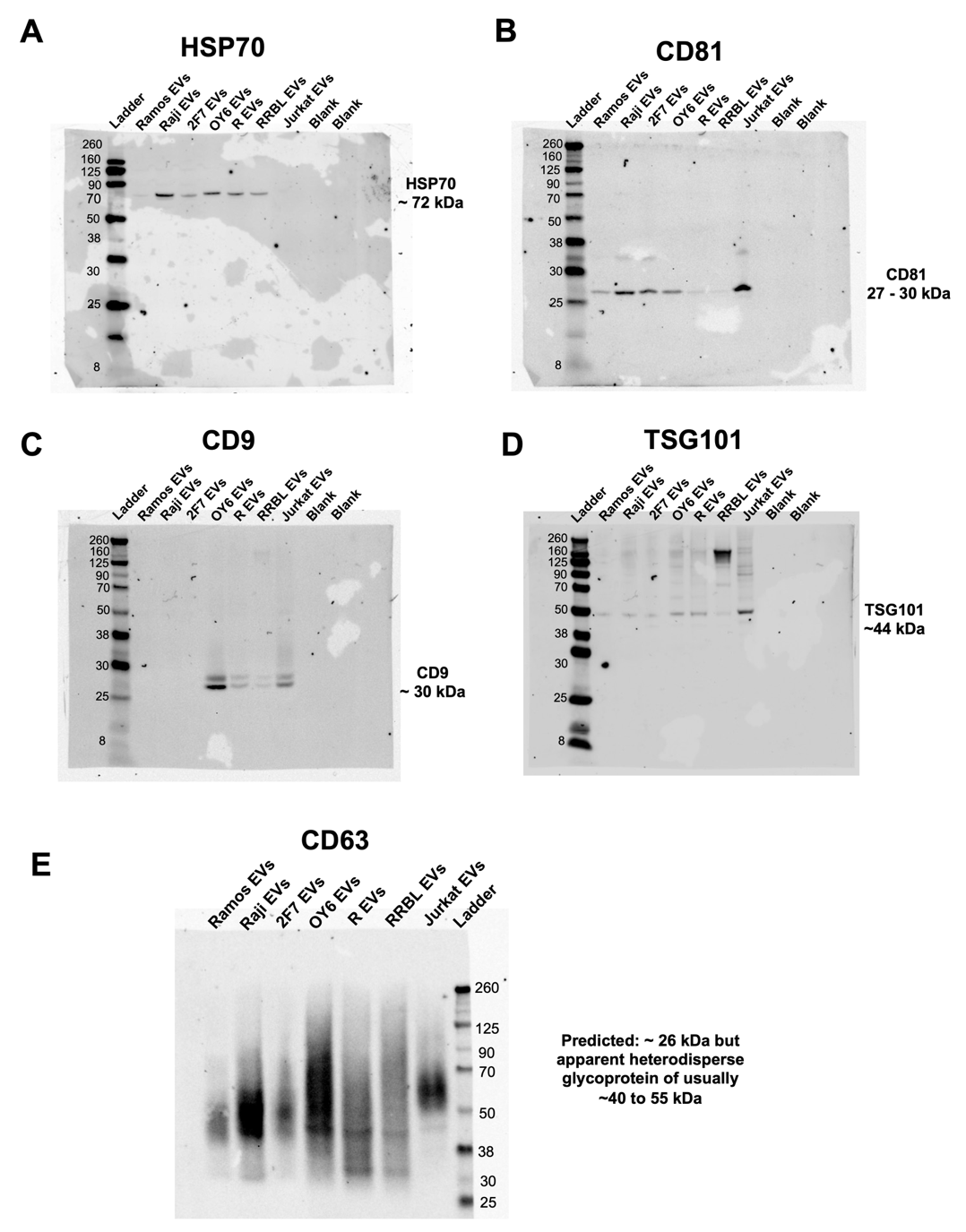
**

**Supplemental Figure 1.** Full-length blots of cropped blots presented in **Figure 3**. (**A, B**) HSP70 (~72 kDa) and CD81 (27-30 kDa) were multiplexed for the same blot. (**C, D**) CD9 (~30 kDa) and TSG101 (~44 kDa) were multiplexed for the same blot. (E) Single target detection for CD63 (predicted: 26 kDa with an apparent heterodisperse glycoprotein of usually 40 to 55 kDa). 20 µg of EV protein lysate was loaded into each well. Imaging/exposure times of each blot: CD81, 60 sec; HSP70, 75 sec; CD9, 60 sec; TSG101, 40 sec; and CD63, 60 sec.
